# Biomarker-Based Signature of Alzheimer’s Disease in Pre-MCI Individuals

**DOI:** 10.3390/brainsci9090213

**Published:** 2019-08-23

**Authors:** Elena Chipi, Nicola Salvadori, Lucia Farotti, Lucilla Parnetti

**Affiliations:** Center for Memory Disturbances, Lab of Clinical Neurochemistry, Section of Neurology, Department of Medicine, University of Perugia, 06132 Perugia, Italy

**Keywords:** preclinical AD, pre-MCI, biomarkers, neuropsychological assessment, neuropathological findings

## Abstract

Alzheimer’s disease (AD) pathology begins decades before the onset of clinical symptoms. It is recognized as a clinicobiological entity, being detectable in vivo independently of the clinical stage by means of pathophysiological biomarkers. Accordingly, neuropathological studies that were carried out on healthy elderly subjects, with or without subjective experience of cognitive decline, reported evidence of AD pathology in a high proportion of cases. At present, mild cognitive impairment (MCI) represents the only clinically diagnosed pre-dementia stage. Several attempts have been carried out to detect AD as early as possible, when subtle cognitive alterations, still not fulfilling MCI criteria, appear. Importantly, pre-MCI individuals showing the positivity of pathophysiological AD biomarkers show a risk of progression similar to MCI patients. In view of successful treatment with disease modifying agents, in a clinical setting, a timely diagnosis is mandatory. In clinical routine, biomarkers assessment should be taken into consideration whenever a subject with subtle cognitive deficits (pre-MCI), who is aware of his/her decline, requests to know the cause of such disturbances. In this review, we report the available neuropsychological and biomarkers data that characterize the pre-MCI patients, thus proposing pre-MCI as the first clinical manifestation of AD.

## Literature Search: Selection Criteria

To obtain studies that specifically include individuals not yet fulfilling MCI criteria, we entered in PubMed database the terms “preclinical Alzheimer’s disease”, “pre-MCI”, “Subtle cognitive decline”, “Subjective cognitive decline”, “Subjective memory decline”, “Subjective cognitive complaints” “Subjective cognitive impairment”, “Subjective memory complaints”, “Subjective memory impairment”, “neuropathology”, “CSF”, “Amyloid PET”, “FDG-PET”, and “MRI”. Our search covered articles that were published between January 2011 and June 2019. 

## 1. Introduction: the Complicated Picture of What Precedes Mild Cognitive Impairment

Pathological aging may be characterized by cognitive and/or behavioral disturbances, often related to neurodegenerative disorders with dementia. Neurodegenerative dementias, the most commonly represented by Alzheimer’s disease (AD), are accompanied by a progressive cognitive decline, which finally leads to loss of functional independence. AD has been conceptualized for a long time as a dual clinicopathological entity, on the basis of a clinical phenotype (amnestic syndrome of hippocampal type) and neuropathologic signs of AD pathology [1]. In the past, the diagnosis of AD was posed when the dementia stage was reached, and it was only based on clinical picture and the exclusion of other causes [2]. Nowadays, this attitude is going to be abandoned, based on the well-established knowledge of dynamic pathophysiological processes detectable in vivo, with the support of core biomarkers (biomarker-based approach) [1,3,4,5]. This led to a conceptual shift from a clinicopathological to a clinicobiological entity, allowing a redefinition of AD as a continuum. A research framework recently proposed a classification model that is based on a categorical classification of biomarker positivity (A/T/N): “A” refers to amyloid pathology (Aβ42 or Aβ42/Aβ40 ratio in cerebrospinal fluid (CSF) or Positron Emission Tomography (PET) scan with amyloid tracers—amyloid PET); “T” refers to tau pathology (CSF phospho-tau or tau-PET); and “N” refers to neurodegeneration (CSF total tau, Magnetic Resonance Imaging (MRI), or fluorodeoxyglucose (FDG)-positron emission tomography (PET) ) [5]. According to this model, AD is defined by biomarkers evidence of cerebral amyloidosis (A+) and tauopathy (T+). Other possible pathologic features may coexist (i.e. Lewy body disease, vascular brain injury, hippocampal sclerosis, argyrophilic grain disease, and TDP-43 inclusions), and these can influence the cognitive outcome [6]. In the A/T/N system, these co-pathologies may contribute to neurodegeneration, and specific biomarkers need to be further validated (i.e. synucleinopathies and primary tauopathies biomarkers). This could lead to extending the use of the ATN system to other neurodegenerative disorders, which improves the diagnostic tools.

Several attempts have been carried out in order to define the pre-dementia phase of AD. Up to now, the only well-recognized symptomatic pre-dementia stage is represented by “Mild Cognitive Impairment” (MCI). In the original definition, MCI was characterized as a heterogeneous clinical syndrome, in which cognitive deficits not fulfilling dementia criteria might be representing a transitional phase from normal cognition to dementia [7,8]. In the biomarker era, the NIA-AA research group defined MCI as “due to AD” in the presence of biomarkers positivity [9]. According to the International Working Group (IWG), this transitional phase was defined as prodromal AD [10]. 

Currently, an increasing number of papers are focusing on the phase preceding the MCI, i.e. “preclinical phase”. Sperling and colleagues [11] proposed three stages for preclinical AD. Stage 1 is characterized by asymptomatic cerebral amyloidosis that was documented either by a positive Amyloid PET or decreased CSF Aβ42 levels; stage 2 by amyloidosis plus neurodegeneration, documented either by hypometabolism at FDG-PET or by increased CSF tau levels or medial temporal lobe atrophy at MRI; stage 3 by evidence of amyloidosis, neurodegeneration, and subtle cognitive decline that does not meet yet the MCI criteria. A recent meta-analysis showed that the risk of progression increases across the preclinical AD stages, where Stage 3 shows a risk of progression that was comparable to MCI due to AD [12]. Accordingly, this phase that is characterized by subtle cognitive decline plus AD biomarker positivity, namely pre-MCI due to AD, might represent the earliest clinical phase of the disease, not actually a preclinical phase. Individuals at the pre-MCI stage of AD need to be further characterized by means of biomarkers, since the combined use of clinical-neuropsychological criteria and AD biomarkers might improve the sensitivity in measuring clinical progression. A biomarker-based classification of pre-MCI individuals is also important in the perspective of a best chance for success of treatment while using novel therapeutic drugs. 

### 1.1. Pre-MCI: Conceptual Evolution and Clinical Issues

Several attempts have been carried out in order to outline the picture of the symptomatic debut of AD, but no shared definition is available at present. Many different lexical terms have been used to refer to this preclinical stage for which no common diagnostic criteria still exist. Among them, “pre-MCI” term has been commonly used to define individuals with subtle cognitive impairment (not still fulfilling MCI criteria). Pre-MCI was originally classified, according to conventional MCI criteria, but with a minor level of cognitive impairment [13]. After the introduction of preclinical AD criteria [11], the concept of “subtle cognitive decline” has been introduced. Anyway, it is not clear whether this term refers to current cognitive status or to longitudinal, intra-individual cognitive changes over time with respect to his/her own baseline level of cognition [14]. Thus, it might be interesting to further focus on this issue. In 2011, Duara and colleagues identified a specific group of pre-MCI patients that were characterized by clinical, neuropsychological, and biological profile [15]. The original conceptualization of pre-MCI was agnostic to the underlying etiology of the clinical picture, and the use of biomarkers was not required, since pre-MCI defined essentially defined as a clinical syndrome. 

Whether pre-MCI category can or cannot be staged as an earliest clinical manifestation of AD is not clear. In fact, like MCI, the pre-MCI category only represents a clinical and heterogeneous syndrome at increased risk for progression to dementia. In absence of biomarkers investigation, little is known regarding the etiology of this syndrome. Accordingly, some pre-MCI patients may have other non-AD pathologies, as well as no specific causes [16]. Thus, the support of pathophysiological biomarkers assessment is recommended in order to rule out AD etiology. In this perspective, individuals with evidence of subtle cognitive decline and AD biomarkers positivity may be diagnosed as “pre-MCI due to AD”, thus representing the earliest clinical manifestation of AD. 

A feasible indicator of a preclinical stage of AD may also be the presence of subjective complaints of cognitive disturbances. Several definitions have been used in the past years, referring to memory/cognitive complaints (Subjective Memory Complaints, SMC; Subjective Cognitive Complaints, SCC; Subjective Memory Impairment, SMI; and, Subjective Cognitive Impairment, SCI). Recently, a research framework aimed at disentangling the heterogeneity among studies and reaching a consensus regarding terminology. This has been made by replacing the heterogeneous terms previously used with the operationalization of the concept of Subjective Cognitive Decline (SCD). According to the definition that was given by the Subjective Cognitive Decline Initiative working group (SCD-I), subjective cognitive decline refers to a self-experienced decline in cognitive functioning with respect to a previously normal status, despite a performance within the normal ranges on standardized cognitive tests [17].

Subjective complaints may be related to several different conditions, such as normal aging, personality traits, psychiatry conditions, neurological and medical disorders, substance use, and medication, and they do not necessarily imply an underlying neurodegenerative cause. However, it has been proposed that the presence of subjective complaints might be associated with lower performance on challenging cognitive tasks [18,19,20,21] and with subsequent cognitive decline and progression to dementia [18,22,23,24,25,26,27], particularly when concerns and worry are reported [19,22,28]. Recently, a conceptual and practical distinction between complaints and worry (or concerns) has been proposed: although a high percent of complaints are reported in older population, a specific concern regarding these feelings of decline may be decisive to move to a clinical consult, and self-reported worry seems to hold a worse prognostic value and it may associated with higher rates of amyloid markers positivity [28]. When SCD is accompanied by evidence of biomarkers positivity, namely “SCD-plus”, this entity may represent an earliest phase of the AD continuum [17] and cognitive performance on neuropsychological assessment declines more steeply [26,29,30]. Accordingly, AD can be ruled out when SCD is not associated with AD biomarkers positivity. However, other pathological conditions might underlie SCD and this issue should be further investigated [1]. Recently, the potential value of SCD assessment to predict and track cognitive decline has been underlined. Amariglio and colleagues suggested that the longitudinal assessment of SCD in individuals with positivity of AD biomarkers might be particularly important for tracking progression of the earliest symptomatic changes of AD [26]. Overall, the boundary between SCD and MCI should not be considered to be a well-defined line, but as a “grey zone”, possibly indicating a pre-MCI stage. When considering that such a subtle, but detectable, impairment may exist in the SCD patient, it has been suggested that SCD could represent the self-perception of these subtle decline before the impairment reaches the threshold for MCI, which indicates the first symptomatic manifestation of the late-stage preclinical AD [21,28,31]. In this sense, SCD can be considered to be a symptomatic indicator of the preclinical AD Stage 3 [32]. Anyway, the presence of subjective cognitive complaints should not be considered a proxy for preclinical AD [1], because they can or cannot accompany the subtle deficits that characterize the phase preceding the MCI. Thus, the term "subtle cognitive decline", as opposed to "subjective cognitive decline", might be more appropriate for subjects who display longitudinal, intra-individual changes over time with respect to a baseline level of cognition, regardless of the presence of subjective cognitive complaints.

In conclusion, individuals with subtle cognitive changes and/or SCD need to be further characterized by means of biomarkers, since they may be in a pre-MCI stage of AD. The need for a better definition of what represents a significant, even if subtle, cognitive, is a current issue and it represents an urgency for the clinical practice, in order to offer a window of opportunity for a timely, disease-modifying therapeutic intervention.

### 1.2. Neuropathological Studies

AD may be pathologically proven at post mortem examination in cognitively healthy individuals, independently on the in vivo detection of AD pathophysiology by means of biomarkers. Some studies reported that between 8% and 45% of cognitively normal adults were found to show AD-related pathology at post-mortem examination [33]. These individuals have been also reported to show limited hippocampal tau and neocortical β-amyloid deposition with respect to those with clinical dementia, who showed more severe, widespread AD pathology [34]. Longitudinal clinico-pathological studies showed that up to 45% of non-demented individuals would meet the criteria for AD, as established in the NIA-RI consensus (1997), especially the intermediate likelihood category [35]. Since the NIA-RI criteria (1997) required both postmortem confirmation of AD and clinical condition of dementia, an updating and revision of these criteria was made in 2011. Along with the criteria for preclinical and clinical AD, neuropathological NIA-AA guidelines were proposed, regardless of the clinical status at decease [6]. Accordingly, AD neuropathological changes can be detected in individuals that were free of cognitive impairment. These subjects have been defined as pathologically preclinical AD [36,37]. 

In the last years, several longitudinal studies in cognitively normal (CN) individuals have been carried out to investigate the correlations of subtle cognitive decline with post mortem evidence of AD. Autoptic confirmation of AD pathology has been associated with in vivo evidences of subtle longitudinal decline in different cognitive domains [38,39]. These subjects may exhibit very early stages of Aβ plaques (1-3), which are not detectable by in vivo PET imaging [40]. 

Price and colleagues [41] previously reported neuropathological findings of AD in 40% of CN individuals showing a subtle decline on multiple cognitive measures (episodic memory, category fluency, global cognition, and divided attention). Riley and colleagues documented that approximately 26% of CN individuals in their cohort met criteria for AD pathology at autopsy [42]. These subjects showed steeper rates of decline with respect to those with no evidence of post mortem AD pathology. Evidences of hippocampal neuritic plaques (NPs) count at autopsy were significantly associated with antemortem longitudinal decline on measures of Global Composite Scores (GCS) and episodic memory, while the combined entorhinal and CA1-hippocampal amyloid load, which expresses a different neuropathological concept, was significantly associated with subtle impairment in speed processing [43]. Boyle and colleagues reported that CN individuals showed different, specific trajectories in the subtle decline on episodic memory, working memory/speed of processing, and semantic memory [38]. Monsell and colleagues found that slopes of changes on longitudinal measures of attention/working memory, rather than episodic memory, were strongly associated with the post mortem confirmation of AD [44].

The NIA-AA conceptual framework regarding the preclinical phase of AD has led to the refinement of the neuropathological criteria for AD [6]. An attempt to validate the preclinical AD three-Stage model with a neuropathological confirmation has been made by Jicha and colleagues [45]. In their study, the authors considered 44 preclinical AD individuals who were cognitively normal at the last neuropsychological evaluation (within one year before the autopsy). They found that 18% of them were classifiable in the Stage 1, 27% in the Stage 2, and the most (55%) met the criteria for the Stage 3. It is noteworthy that all of the subjects were cognitively intact until the decease, but subtle changes on Mini-Mental State Examination (MMSE) was noted in those in the Stage 3 as compared to the others; moreover, the most common pathology burden met the Braak III-IV stages, which means no significant neurofibrillary accumulation. It is interesting that a recent study reported that individuals that are classifiable in the NIA-AA preclinical AD Stage 2 and 3 did not differ from MCI patients in terms of amyloid positivity, while they showed higher SUVRs within specific subcortical regions (putamen and accumbens) with respect to those in Stage 0 and 1 [46]. This novel finding may have impact on the knowledge regarding the correspondence of the in vivo and the post-mortem neuropathological hallmarks distribution.

Memory complaints in CN individuals may predict the presence of AD neuropathological changes [47]. The rates of memory complaints correlated with changes in brain AD neuropathological changes in a linear manner, and remained significant after controlling for depressive symptoms. Recently, Kryscio and colleagues analyzed whether neuropathological findings can be detected in individuals with subjective cognitive decline (SCD). Their results underlined that SCD subjects who later converted to dementia showed accelerated the annual slope of decline on measures of episodic memory and naming. At the autoptic examination, they displayed clear AD pathology. Complainers who did not convert to dementia showed post mortem evidence of amyloid and tangles deposition, but at a minor level of magnitude when compared to converters [48]. Previously, they found that, among 176 participants who died without clinical impairment, memory complaints were associated with elevated neuritic amyloid plaques burden in the neocortex and in the medial temporal lobe [49]. 

Overall, these neuropathological studies highlighted that individuals with SCD may share the same pathophysiology of clinically AD individuals, and they may be placed in an intermediate stage between normal aging and clinical AD within the Alzheimer’s continuum.

## 2. Pre-MCI: Neuropsychological and Biomarkers Findings

The current study reports growing literature regarding evidences of biomarkers AD-signatures in pre-MCI individuals. Specifically, we analyzed neuropsychological and biomarkers profiles focusing on studies that have considered participants with: subtle cognitive impairment (i.e., those who may show subtle deficits at baseline who did not reach neuropsychological criteria for MCI) and/or subtle cognitive decline (i.e., those who may show subtle longitudinal worsening) and/or subjective cognitive decline, as defined by Jessen criteria [17].

### 2.1. Neuropsychological Characterization

The identification of neuropsychological measures that are sensitive enough to detect early subtle cognitive changes related to AD pathology is crucial in order to timely intervene with novel disease-modifying drugs [50]. In the section below, we discuss each term composing the concept of “subtle cognitive decline” [11].

#### 2.1.1. Subtle Cognitive Decline: What “Subtle” Stands For

Subtle means that there is the presence of a cognitive impairment, but this does not yet meet the MCI criteria. To define when a cognitive performance falls into pathological scores is a controversial issue, and heterogeneous criteria have been adopted. For example, Loewenstein and colleagues [16] classified the subjects who do not fulfill criteria for MCI in three subtypes of pre-MCI, defined by the number of impaired neuropsychological tests and the cut-off used to define this impairment. A cut-off less than −1.25 SD (lowest tenth percentile of the distribution in the group) in episodic memory composite score and a Clinical Dementia Rating (CDR) score of 0 were applied to signify subtle cognitive decline in preclinical AD stage 3 [51]. Edmonds and colleagues suggested that subtle cognitive decline has to be characterized by a score >1 SD below the age-corrected normative mean on at least two of the six neuropsychological measures used, in different cognitive domains [52]. The importance of also focusing on other cognitive domains beyond memory to better detect subtle alterations has been underlined in a recent review of Epelbaum and colleagues, who proposed the concept of “Preclinical AD with subtle cognitive changes”. They introduced novel guidelines for operationalizing this early stage of AD, in which patients display performances of less than 1 SD in one or more cognitive measures, in the absence of more specific symptoms [53]. 

In clinical setting, the need to define a standard cut-off for subtle cognitive impairment is crucial to avoid mis-diagnosis. This is particularly true for those individuals potentially at risk for incipient AD, who might take advantage from novel prevention trials.

#### 2.1.2. Subtle Cognitive Decline: What “Cognitive” Stands For

Cognitive means that there is a poor performance on more challenging cognitive tests, therefore it mandatory to consider specific highly demanding tasks. It is crucial to develop novel paradigms that are (1) sensitive to detect early cognitive symptoms of the disease, (2) specific in the differentiation from normal performance, (3) associate with biomarkers, and (4) not susceptible to individual variability in learning strategies or compensatory mechanism that may mask subtle deficits [16]. Among various attempts, associative bindings tasks, composite scores, or computerized measures seem to be the most promising paradigms.

Several studies recently found that a very sensitive measure to early detect subtle cognitive decline is represented by the associative learning paradigm: in particular, the Free and Cued Selective Reminding Test (FCSRT) is a multimodal associative memory measure, in which learning is enhanced by a semantic category cue [30,54]. This paradigm is distinguishable by the other word list memory tasks, in which each subject might properly develop a strategy for encoding; conversely, the cued memory FCSRT task rules out the confounding variable that is represented by executive processes underlying effective learning strategy, and it inherently points to mnemonic processes [55]. Moreover, the association between words and pictures may recruit a widespread neural network that involves not only the medial temporal regions, but also the prefrontal cortex, and the interplay between the frontal regions and hippocampal and parahippocampal cortices [56]. Some evidences suggest that the immediate recall, rather than delayed recall, may be the earliest measure detecting subtle deficits in the preclinical AD phases. Accordingly, individuals with Aβ positivity alone may show a subtle decline in the FCSRT-free recall, as an effect of brain amyloidosis within prefrontal regions, whereas the FCSRT-total recall seems to be more dependent by the temporo-limbic cortices, and a subtle decline in FCSRT-TR can reflect tangle pathology within these regions [55]. 

Overall, these data revealed that, contrary to the original proposal of the NIA-AA Preclinical AD Stages model, the individuals in Stage 2 may already display subtle deficits, some of them may be present yet in the Stage 1, and the co-occurrence of amyloidosis and neurodegeneration may represent the highest risk for developing cognitive decline. 

The FCSRT is suitable for the detection of subtle cognitive decline, especially when included in composite scores, encompassing other cognitive tests. These novel neuropsychological combined measures proved to have higher sensitivity than single cognitive tests in detecting decline during preclinical AD continuum [25,26,57,58,59,60]. Accordingly, they have been introduced as the outcomes of preclinical trials [58,59,60,61], due to their suitability in tracking the subtle cognitive changes that can occur in the preclinical AD phases. For instance, a specific composite score, namely PACC (Preclinical Alzheimer Cognitive Composite), was recently shown to be able to capture Aβ-related cognitive decline and progression to functional impairment, as assessed by the Clinical Dementia Rating scale [30]. 

Other neuropsychological measures that can be promising to capture subtle decline in preclinical AD are represented by visual tasks from computerized batteries. They provide frequent serial measurements of intra-individual performance over brief time intervals. They may also be suited for use in detecting subtle longitudinal changes in clinical trials targeting clinically normal adults that are at risk of AD-related cognitive decline [57,62]. These batteries include reaction-timed tests, continuous monitoring and working memory tasks, as well as associative learning tasks. Some evidences supported the usefulness of a specific computerized measure from the Cambridge Neuropsychological Test Automated Battery (CANTAB) [63], the Paired Associates Learning (PAL). The PAL is a computerized measure that is particularly sensitive to the medial temporal lobe dysfunction, as proven by clinical and neuroimaging studies on MCI patients [64]. In recent years, several studies documented the suitability of the PAL as a neuropsychological marker of early AD cognitive changes, and its reliability as a tool for differential diagnosis has been proved in cross-sectional studies [65,66]. A recent study revealed that individuals with subjective cognitive decline who reported poor performances on the PAL showed abnormal CSF p-Tau levels that were up to 10 years before [67], which supported the hypothesis that this measure reflects medial temporal lobe (hippocampal and parahippocampal) dysfunction due to neurofibrillary tangles deposition, as mirrored by the p-Tau CSF levels. 

#### 2.1.3. Subtle Cognitive Decline: What “Decline” Stands For

Decline evidences a cognitive change with respect to a previous state of cognitive functioning. A decline in the cognitive test scores could begin many years before dementia onset. Nevertheless, the test scores may continue to remain within the average limits for age, masking the decline. Accordingly, it is mandatory to use measures that are able to capture intra-individual cognitive decline, even if still within a “normal” range during the preclinical phase. Consistent findings while using composite scores in previous cohorts confirmed that the combination of cognitive domains, rather than single isolated tests, is particularly suitable to detect longitudinal decline during the stages preceding MCI [30]. Single assessment cannot capture these early subtle cognitive changes; thus, it is mandatory to follow the patient with repeated observation over time, while considering the patient as a control of him- or herself. In a recent paper, it has been found that the identification of neuropsychological decline, beyond a single cognitive evaluation, improved prognostic accuracy, even in asymptomatic older adults [68]. Conceptually, these findings are in line with the notion that there is the existence of a neuropsychological performance continuum that spans from the normal to MCI stage [5,68]. Thus, detecting clinically significant cognitive change in the early preclinical stages of AD is challenging. Serial neuropsychological assessment might be the favored method for evaluating the cognitive decline. 

A reliable source of estimated intra-individual change is the informant report from the CDR; a score of 0.5, which was common for MCI and pre-MCI patients, can track the amount of functional—rather than cognitive—decline with respect to a previous level. Notably, the CDR-Sum of Boxes (CDR-SoB) is currently included in the composite scores as a useful measure for tracking subtle changes in the preclinical AD phases [69]. The importance to consider clinical changes—as reflected by CDR scores—besides the neuropsychological measures has been documented. In fact, distinct phenotypes of pre-MCI were found, on the basis of both cognitive and clinical decline. Pre-MCI with CDR of 0.5 plus evidence of impairment in two or more memory tests were more prone to progress over time [16]. The authors recommended carrying out long follow-up observations in order to clarify the clinical outcome of these patients.

Regarding SCD individuals, it is important to identify instruments that are able to define and quantify the subjective perception of decline, because this entity has been frequently reported as a potential indicator of AD, especially when associated with biomarkers positivity [17,28,32,70]. Standardizing measurements of SCD is challenging, due to the subjectivity of self-reported feelings. However, self-report measures remain the most commonly used instrument and it may be particularly useful to track early decline [31]. For example, increased scores on Cognitive Function Instrument (CFI) [71], a questionnaire that covers cognitive and functional changes, predicted higher CDR scores in the absence of objective cognitive impairment, as detectable by neuropsychological tools. Its use for tracking early cognitive decline should be taken into account in lengthy prevention trials, including cognitively normal individuals [72].

KEY POINTS: Detecting subtle cognitive impairment not yet classifiable as MCI is crucial to lead to the early diagnosis of AD. Nowadays, some heterogeneity about neuropsychological criteria used still persists, and an agreement is not reached. Clinical profiles that are characterized by very subtle decline not meeting standard criteria for MCI may deserve to be further explored with more advanced tools sensitive enough to detect slight cognitive deficits. Unconventional neuropsychological measures, as composite measures and computerized test, might be a useful approach to improve diagnostic sensitivity in the detection of subtle cognitive decline, especially when longitudinally repeated.

### 2.2. Pathophysiological Biomarkers

In the present section, we report current evidences about pathophysiological/diagnostic biomarkers (CSF and amyloid-PET) positivity in pre-MCI patients. Table 1 summarizes a selection of articles that reported data about clinical progression in these individuals.

#### 2.2.1. CSF

A cerebrospinal fluid (CSF) profile that is compatible with AD is represented by evidence of clearly alterations in all the three core AD biomarkers, namely β-amyloid 1-42 (Aβ42), total tau (t-tau), and phospho-tau (p-tau). Recently, Aβ40 has been included in the panel of diagnostic AD biomarkers [77,78], since several evidences reported that the Aβ42/Aβ40 ratio outperforms Aβ42 alone [79,80,81,82], and the ratio showed better concordance with amyloid PET positivity [83].

Early CSF alterations are the first altered in vivo biological markers of AD [84,85,86]. It is noteworthy that, according to the current NIA-AA research framework proposal, the only presence of β-amyloid alteration (either in CSF or as positive PET scan) is defined as “AD pathologic change” in the AD continuum. Authors recommended defining “preclinical AD” aw the co-presence of abnormal Aβ42 (or Aβ42/Aβ40) and p-tau [1,5].

The rates of CSF positivity in preclinical AD individuals are heterogeneous, depending on the criterion that is used to define the CSF AD-signature (global profile, Aβ42/tau or Aβ42/p-tau ratio, statistical formulas). In SCD individuals, the prevalence ranged from 7–24% while using descriptive or statistical algorithms [87] or while considering the whole profile [32,88,89], reaching 34–55% using the Aβ42/tau ratio [90]. When compared to MCI, the SCD individuals showed significantly lower levels of t-tau and p-tau, whereas differences in the Aβ42 levels were less pronounced [91]. Furthermore, a recent study reported significantly lower levels of Aβ42 in SCD than the controls, despite similar levels of t-tau and p-tau [92]. In a recent paper, 46% of SCD displayed brain amyloidosis, whereas 18% of them showed preclinical AD (A+T+) at CSF profile [93].

Evidences are concordant in reporting that cognitive impairment is significantly associated with Aβ42 in the early phases of AD, whereas the association is more robust with tau and p-tau in the later, MCI phase [91,94,95]. In SCD, low Aβ42 was associated with worse scores on the measures of verbal and visual episodic memory [91,94], semantic memory, and working memory [95]. In a study of Grambaite and colleagues, a non-memory measure—as inhibitory control—showed a trend in correlation with the CSF biomarkers, and they suggested that this could be an early neuropsychological marker for detecting a subtle decline in these SCD individuals [91]. Similar results were described by Harrington and colleagues, who reported an association between Aβ42/tau ratio and performances on the Stroop test in preclinical AD individuals [85]. Overall, these data are in line with the hypothesis that an early impact of brain amyloid deposition on executive functions can precede memory changes, in preclinical AD [53,96].

Regarding the prognostic value of CSF biomarkers, several authors reported that Aβ42 is the best marker to predict cognitive progression in SCD individuals [75,76,94,95]. However, this evidence is still controversial. Many studies found a preeminent role of the Aβ/tau ratio in predicting cognitive impairment over time [79,97,98], and others described as the global CSF profile alteration at baseline, namely both pathological Aβ42 and tau values, holds the highest risk for conversion [84,98,99]. Recently, Wolfsgruber and colleagues reported that 22% of SCD progressed to MCI at two-year follow-up, but prevalence the reached 60% in those who showed of AD-like CSF profile [32].

KEY POINTS: CSF Aβ42 reduction is associated with subsequent cognitive impairment in individuals with SCD, especially on measures of episodic memory and executive functions, while the CSF t-tau and p-tau values correlate with cognitive performance only at later (i.e., prodromal) stages. However, a globally altered CSF profile stronger correlates with the entity of cognitive decline, even in SCD patients.

#### 2.2.2. Amyloid PET

Cognitively normal (CN) individuals, with or without subjective cognitive complaints, can display brain amyloidosis (Aβ+) at Positron Emission Tomography (PET) scan with amyloid tracers (amyloid PET). The prevalence of amyloid PET positivity ranges from 10 to 40% in CN [100]. Some authors documented that subjective cognitive concerns are associated with a higher prevalence of amyloid PET positivity [101,102,103,104], while others did not find any difference in rates of positivity between SCD and controls [100,105]. A recent review and meta-analysis replicated this finding, reporting analogous rates of amyloid PET positivity (22–23%) in SCD and CN [12]. 

Some studies reported evidence of subtle cognitive impairment in cognitively normal (CN) individuals with amyloid PET positivity as compared to those with negative scan [74,106], while others did not find the same results [73,107]. Chètelat and colleagues [108] found that, in CN individuals with memory concerns, amyloid uptake in the temporal neocortex was associated with subtle episodic memory deficits independently on hippocampal atrophy. Previous studies found a significant association between Aβ+ and episodic memory performance at the baseline [109], while a recent study confirmed such an association only after 70 years [107]. 

More robust associations were found between amyloid positivity and longitudinal decline at follow-up observation, but what cognitive measures are involved is less clear. Studies reported a specific decline on episodic memory [74,102,110,111], others found an impact on multiple measures [14,105,106,111,112], especially when the composite scores were used [14,30]. Timmers and colleagues found that SCD with PET positivity had a trend in displaying subtle cognitive deficits at baseline, but a significant cognitive decline occurred at longitudinal assessment [113]. Conversely, Dubois and colleagues found that SCD individuals with amyloid PET positivity did not show any subtle cognitive impairment at baseline or at 30-month follow-up [73]. However, the length of follow-up should be taken into consideration, since two studies documented longitudinal decline in SCD Aβ+ subjects on episodic memory and global cognition [114], and on composite scores [26]. A recent study found that SCD with Aβ+ showed higher decline on a composite scores at four-year follow-up [26]. Interestingly, rates of cognitive concerns also increased over time, as a support of an alignment of subjective and objective decline in the early phases of AD [71]. Conversely, others did not find a significant longitudinal increase in the memory concerns in Aβ+ individuals, despite evidence of episodic memory decline occurring [105]. Vogel and colleagues [114] documented the synergistic effect of SCD and amyloid positivity in predicting higher rates of decline on episodic memory and global cognition; as an interesting finding, worse SCD predicted worse scores on the measures of working memory independently from PET status. 

Overall, these results supported the need to consider the impact of both SCD and amyloid status on longitudinal cognitive decline in pre-MCI individuals.

KEY POINTS: The association between amyloid PET positivity and subtle cognitive impairment, mainly being assessed by episodic memory scores, is documented both in cognitively normal and in SCD individuals. The baseline associations of PET positivity with cognitive scores are not clear in these subjects. Conversely, several studies support higher rates of longitudinal cognitive decline, mainly on episodic memory but also in other neuropsychological measures. 

### 2.3. Topographical Biomarkers

In the present section, we report current evidences regarding topographical/progression biomarkers (FDG-PET and MRI) positivity in pre-MCI patients.

#### 2.3.1. FDG-PET

Early studies documented the evidence of alterations in brain glucose metabolism in pre-MCI individuals. A study reported that regional changes in brain metabolism within temporal-parietal and prefrontal areas predicted cognitive decline after three years in cognitively normal subjects [115]. Interestingly, poor scores on executive functions measures, but not on episodic memory, improved the diagnostic accuracy when associated with brain metabolism. Instead, in a cross-sectional study, Brugnolo and colleagues reported posterior cingulate/precuneus hypometabolism in MCI, but not in SCD patients who performed an episodic memory test [116]. A recent paper supported these data, showing no evidence of hyper- or hypometbolism in SCD patients [117]. 

Other studies found evidences of brain metabolic changes in SCD individuals. Scheef and colleagues reported a regional pattern of hypometabolism in right precuneus and left parietal cortex, and hypermetabolism in right medial temporal lobe of SCD patients who later displayed episodic memory decline [118]. Authors concluded that topographical abnormalities in AD-vulnerable brain regions can be detectable in the early phases of the disease. In another recent study, brain hypometabolism was reported in the right amygdala in SCD individuals who performed poorly on measures of immediate verbal recall, but not delayed visual or verbal recall [119]. A previous study reported that individuals with memory complaints, especially if the carriers of APOE ε4 alleles, showed brain glucose hypometabolism in the parieto-temporal cortex and in the parahippocampal gyrus, with respect with cognitively healthy individuals without subjective impairment [120]. The selective decrease in glucose metabolism within the periventricular regions has been documented in SCD when compared to MCI patients, who exhibited more widespread metabolic changes also involving parietal and pre-central areas [121]. No differences were found in any neuropsychological measure. The authors concluded that a widespread brain hypometabolism from periventricular regions toward the involvement of the parietal lobes may represent the transitional metabolic threshold for identifying the progression to MCI stage. In SCD patients with high amyloid deposition, a synergistic effect of Aβ+ and memory complaints on brain metabolism decrease has been found in the medial temporal lobe, especially the hippocampus [104]. Hypometabolism in bilateral precuneus and inferior parietal regions, right inferior temporal lobe, and right frontal lobes was only related to memory complaints, but not to amyloid status. 

Evidences of hyper-metabolism can be detected in SCD individuals that perform poorly in memory tasks: rates of increased brain metabolism have been reported in bilateral insular, right inferior parietal lobe, and fusiform area [122]. Temporo-parietal hypometabolism detectable in the prodromal phases might be preceded by an initial hypermetabolism in the same regions during the preclinical AD, as a consequence of compensatory mechanisms [123]. 

According to the NIA-AA preclinical AD stages, Knopman and colleagues [124] reported that individuals in the Stage 2/3 of the preclinical phase showed higher rates of brain hypometabolism within several medial temporal lobe structures (hippocampus, parahippocampal gyrus, entorhinal cortex, and amygdala) when compared those in Stage 0 at follow-up. 

KEY POINTS: Brain regional metabolic changes can predict cognitive decline up to several years before symptoms onset in cognitively healthy individuals. In SCD, the rates of hypometabolism have been described within temporo-parietal and periventricular regions, and in the right amygdala. Conversely, aberrant brain activation (i.e., hyper-metabolism) can also be found, probably due to compensatory mechanisms during the preclinical phases of the disease.

#### 2.3.2. MRI

Subtle alterations in brain volumes can be detectable in preclinical AD stages [125,126,127,128,129]. In cognitively normal individuals, gray matter volume loss in the medial temporal lobe, precuneus/posterior cingulate, and the orbitofrontal cortex can predict cognitive decline up to 10 years before symptom onset [127]. The entorhinal cortex seems to be involved earlier than hippocampus and amygdala, and it can be predictive of the symptoms onset up to 8–10 years before [130,131]. 

A pool of temporo-parietal regions has been widely described as an AD-vulnerable network, in which the earliest structural changes of preclinical AD occur. This widespread brain network involves the hippocampus, precuneus/posterior cingulate, inferior and superior frontal cortex, temporal pole, inferior and superior temporal cortex, the angular gyrus, and the sovramarginal gyrus [132,133]. Structural abnormalities in these regions represent a marker of AD pathology, so-called “AD-signature” [134,135]. The regions have been previously described as the Default Mode Network (DMN) [136,137,138,139]. The structural abnormalities in DMN resemble the same brain distribution of amyloid deposition in AD. Recent findings reported early abnormalities in the hippocampal formation [130] and subfield abnormalities [140] during the preclinical phase of the disease. Accordingly, subjects in the stage 2/3 of NIA-AA preclinical phase of AD might present accelerated atrophy in the medial temporal lobe structures [141].

In agreement with the recent A/T/N model [4,5], regional patterns of brain atrophy that are driven by AD pathology are evident when both low Aβ42 and high p-tau CSF levels are found. Some studies support the evidence that markers of amyloidosis (Aβ42, A+) and tangles pathology (p-tau, T+), but not injury markers (t-tau, N+), are necessary for brain volume changes that are detectable in early phases [142,143,144]. 

Several studies reported that individuals with SCD show gray matter volume reduction or cortical thinning in different brain areas [118,131,140,145,146]. Supporting the hypothesis of SCD as an early AD phase, Perrotin and colleagues found significant volume reduction in hippocampal subfields (CA1 and subiculum) in SCD individuals when compared to controls [140]. Recently, a study confirmed the evidence of lower volumes in left hippocampus, CA1, subiculum, and fibria in SCD individuals [147].

Significant thickness reduction in the entorhinal cortex was documented in individuals with SCD as compared with controls [146]. Furthermore, Schultz and colleagues reported significant thinning in the posterior cingulate, inferior parietal lobule, fusiform gyrus, and amygdala in SCD patients [131]. Thinner volumes in a parieto-temporal pool of brain areas as part of the “AD-signature” predict a higher risk to progress to MCI or dementia within one year, especially when the SCD patients also showed preclinical AD (A+T+) at CSF biomarkers [148]. 

In recent years, advanced techniques of structural and functional connectivity have been applied to study the early brain changes in SCD. Increased functional connectivity and volume reductions in regions of the DMN were found in SCD individuals [149]. Ryu and colleagues found low fractional anisotropy and increased mean diffusivity in the head of hippocampus and in the white matter of entorhinal cortex in SCD when compared to the controls [150]. Lastly, altered coherence in the cortical connectivity of precuneus and fronto-occipital network has been associated with steeper cognitive decline in these individuals [151]. 

Taken together, these findings support evidence that early macro- and micro- structural brain changes are both detectable in pre-MCI individuals with preclinical AD and/or SCD. 

KEY POINTS: Subtle structural changes in specific brain areas are detectable during the preclinical AD phase. Individuals with SCD may show volumetric reduction or thinning in the entorhinal cortex, in hippocampus and its subfields. However, other parieto-temporal brain regions show early abnormalities in SCD individuals. A recent approach suggested considering the subtle changes that occur in a large-scale network of vulnerable brain areas, so-called “AD signature”, rather than focusing on alterations in isolated cortical regions. Advanced MRI-structural and functional connectivity measures can help to further investigate the subtle brain changes occurring in the pre-MCI AD phases.

## 3. Discussion

The present work aimed to review the current literature regarding evidences of pathophysiological and topographic biomarkers positivity in pre-MCI, defined as subjects with subtle deficits not fulfilling MCI, in order to analyze the association of such a positivity with cognitive decline. To the best of our knowledge, this is the first bibliographic review focusing on this issue, underlying the impact on research and clinical practice. Our results suggest the possibility to extend the AD biomarkers assessment in subjects with subtle cognitive alterations (pre-MCI), as the earliest clinical manifestation of AD. The Summary box presents a synthesis of the main findings derived from literature review and future implications. Few meta-analyses have been published to investigate the association of AD biomarkers positivity and subtle cognitive deficits in pre-MCI individuals. A previous work found a significant relationship of brain amyloidosis (as assessed by CSF or amyloid PET) with poor neuropsychological scores—still remaining within the normal range—in asymptomatic individuals. They suggested that small, but detectable, cognitive impairment might represent an indicator of amyloid burden that is associated with preclinical AD [110]. An update of this work has been proposed by Baker and colleagues [14], who investigated the magnitude of Aβ-related cognitive changes, considering cross-sectional and longitudinal studies. Overall, in asymptomatic subjects, Aβ+ was associated with both subtle cognitive “impairment” (defined as slight alterations at baseline) and “decline” (defined as slight longitudinal changes over time). Subsequently, a meta-analysis revealed that the combination of amyloidosis and neurodegeneration (A+N+, Stage 2) leads to lower performance on memory domain with respect to only amyloidosis (A+, Stage 1) [152]. A recent meta-analysis [12] found that the risk of progression increases across the preclinical AD stages, where individuals in Stage 3 have the highest risk (73%) when compared to others stages (Stage 2, 38%; Stage 1, 20%) and to those with normal biomarkers (six-fold higher risk). 

Here, we focused on five longitudinal studies reporting the clinical progression in pre-MCI subjects with pathophysiological AD biomarkers positivity (Table 1). Although few studied have been considered, a higher percentage of progression to MCI or dementia was found in subjects with CSF positivity (ranging from 50% to 65%), while a lower percentage was found in subjects with amyloid PET positivity (ranging from 5% to 32%). Heterogeneity in the prevalence of biomarkers positivity and rates of progression among studies could be explained by methodological differences (i.e., inclusion criteria, samples size, duration of follow-up, clinical outcome). Additionally, it is noteworthy that the choice of CSF or amyloid PET determines a significant influence on these results, since the former provides information regarding amyloidosis and tauopathy, while the latter only accounts for the amyloid status.

SUMMARY BOX: Presence of amyloidosis (A+) and tauopathy (T+) allows to formulate Alzheimer’s disease (AD) diagnosis, independent of clinical stage. At present, the earliest clinical stage for diagnosing AD is Mild Cognitive Impairment (MCI) associated with positivity of these biomarkers. In this review, we considered the available clinical and post-mortem studies carried out in individuals with subtle cognitive impairment/subjective cognitive decline, not fulfilling MCI diagnosis (“pre-MCI”), in whom presence of AD was documented.

MAIN FINDINGS: CSF AD-signature/Amyloid PET positivity/AD-like metabolic changes at FDG-PET/temporo-parietal patterns of brain atrophy at MRI can be detected in pre-MCI subjects and are associated with cognitive worsening. 

IMPLICATIONS: Since evidences of AD biomarkers positivity are found in pre-MCI, the accurate characterization of this condition is a major issue. Accordingly, standardized neuropsychological criteria are highly needed. Pre-MCI with AD biomarkers positivity might be the ideal category for treatment with disease-modifying agents, representing the earliest clinical manifestation of AD.

## 4. Conclusions

A growing literature supports the chance to early detect AD even at pre-MCI stages by means of neuropsychological assessment and biomarkers. Current researches are focused on subjective reports of cognitive decline as a putative early manifestation of the disease, since they might represent risk factor for clinical progression. However, SCD per se is not sufficient to be interpreted as a proxy for clinical AD, and its characterization by means of biomarkers is mandatory to clarify the etiology of these symptoms. Recently, based on the NIA-AA criteria for preclinical AD, many efforts have been made to capture subtle (pre-MCI) cognitive deficits in individuals with or without subjective cognitive decline. These subjects can rely on the AD continuum at an early, but yet symptomatic, phase. We propose adopting the term "subtle cognitive decline", as opposed to "subjective cognitive decline", for those subjects who display longitudinal, intra-individual changes over time with respect to his/her own baseline level of cognition, since some evidences have found that preclinical AD populations may show subtle deficits either at baseline or longitudinally. Conversely, we suggest using the term "subtle cognitive impairment" for those individuals with subclinical deficits with respect to their age-adjusted norm, not fulfilling MCI. Both of these two categories of subtle deficits can or cannot be accompanied by subjective cognitive complaints.

A timely diagnosis is mandatory in a clinical setting, in order to offer a window of opportunity to intervene with novel disease-modifying drugs. Moreover, knowing the cause of an individual’s cognitive impairment enables the delivery of timely and appropriate personalized care (i.e. prevent unsafe behaviors and manage symptoms). Thus, it is mandatory to extend AD diagnosis to this earliest—pre-MCI—symptomatic stage.

In conclusion, on the basis of the available evidences of biomarkers positivity in preclinical AD population, it should be recommended to not neglect subjective cognitive decline, poor scores on advanced neuropsychological measures, and intra-individual changes in cognitive performance. In memory clinic, in the presence of these clinical manifestations, biomarkers assessment should be proposed to those individuals who will to know, because it is crucial to rule out an underlying AD pathology.

## Figures and Tables

**Table 1:** Clinical progression in “pre-MCI” subjects with pathophysiological biomarkers positivity.

Reference	Group (N)	Mean Follow-Up	Biomarker Assessed	% Biomarkers+ (N)	% Progress (N)	Clinical Progression
Dubois et al. 2018 [73]	SMC (318)	2 years	Amyloid PET	28% (88)	5% (4)	MCI
Donohue et al. 2017 [74]	CN (445)	3.1 years	Amyloid PET	45% (202)	32% (71)	MCI
Wolfsgruber et al. 2017 [32]	SCD (82)	2.3 years	CSF	24% (20) *	65% (13)	MCI/dementia
Van Harten et al. 2013 [75]	SCC (132)	1.5 years	CSF	7% (10) *	60% (6)	MCI/dementia
Van Harten et al. 2013 [76]	SCC (127)	3.9 years	CSF	7% (10) *	50% (5)	MCI/dementia

CN: cognitively normal; CSF: cerebrospinal fluid; MCI: Mild Cognitive Impairment; PET: Positron Emission Tomography; SCD: subjective cognitive decline; SCC: subjective cognitive complaints; SMC: subjective memory complaints. * abnormal Aβ_42_ + abnormal tau and/or p-tau.
